# *PPIA*, *HPRT1*, and *YWHAZ* are suitable reference genes for quantitative polymerase chain reaction assay of the hypothalamic–pituitary–gonadal axis in sows

**DOI:** 10.5713/ab.22.0083

**Published:** 2022-05-02

**Authors:** Hwan-Deuk Kim, Chan-Hee Jo, Yong-Ho Choe, Hyeon-Jeong Lee, Min Jang, Seul-Gi Bae, Sung-Ho Yun, Sung-Lim Lee, Gyu-Jin Rho, Seung-Joon Kim, Won-Jae Lee

**Affiliations:** 1 College of Veterinary Medicine, Kyungpook National University, Daegu 41566, Korea; 2 Department of Veterinary Research, Daegu Metropolitan City Institute of Health & Environment, Daegu 42183, Korea; 3 College of Veterinary Medicine, Gyeongsang National University, Jinju 52828, Korea

**Keywords:** Hypothalamic-pituitary-gonadal Axis, Sow, Reference Gene, Quantitative Reverse Transcription Polymerase Chain Reaction

## Abstract

**Objective:**

The quantitative reverse transcription polymerase chain reaction (qPCR) is the most accurate and reliable technique for analysis of gene expression. Endogenous reference genes (RGs) have been used to normalize qPCR data, although their expression may vary in different tissues and experimental conditions. Verification of the stability of RGs in selected samples is a prerequisite for reliable results. Therefore, we attempted to identify the most stable RGs in the hypothalamic–pituitary–gonadal (HPG) axis in sows.

**Methods:**

The cycle threshold values of nine commonly used RGs (*18S*, *HPRT1*, *GAPDH*, *RPL4*, *PPIA*, *B2M*, *YWHAZ*, *ACTB*, and *SDHA*) from HPG axis-related tissues in the domestic sows in the different stages of estrus cycle were analyzed using two RG-finding programs, geNorm and Normfinder, to rank the stability of the pool of RGs. In addition, the effect of the most and least stable RGs was examined by normalization of the target gene, gonadotropin-releasing hormone (*GnRH*), in the hypothalamus.

**Results:**

*PPIA*, *HPRT1*, and *YWHAZ* were the most stable RGs in the HPG axis-related tissues in sows regardless of the stages of estrus cycle. In contrast, traditional RGs, including *18S* and *ACTB*, were found to be the least stable under these experimental conditions. In particular, in the normalization of *GnRH* expression in the hypothalamus against several stable RGs, *PPIA*, *HPRT1*, and *YWHAZ*, could generate significant (p<0.05) elevation of *GnRH* in the preovulatory phase compared to the luteal phase, but the traditional RGs with the least stability (*18S* and *ACTB*) did not show a significant difference between groups.

**Conclusion:**

These results indicate the importance of verifying RG stability prior to commencing research and may contribute to experimental design in the field of animal reproductive physiology as reference data.

## INTRODUCTION

Quantitative analysis of gene expression is essential in the field of biology and veterinary research to understand the gene regulatory network. Because quantitative reverse transcription polymerase chain reaction (qPCR) is able to simultaneously compare gene expression in various samples and provides high convenience, sensitivity, reproducibility, accuracy, and reliability, it is considered as the standard method for quantification of gene transcripts. However, the results of qPCR can be critically affected by several factors, including the quality of nucleic acids, amount of starting material, method of RNA preparation, tissue degradation, sampling method, specificity of PCR products, and DNA dye [1,2]. The most common technique used to adjust for these variations is normalization of the expression level of a target gene against constitutively expressed reference genes (RGs) [3,4].

The RGs play a critical role in basic cellular functions and cell survival, including energy generation, substance synthesis, and cell defense/death, and are believed to be stably expressed regardless of environmental and experimental conditions [5]. Unfortunately, there is no single universal RG that is consistently expressed in all experimental situations; accumulated evidence has shown that expression of RGs is variable according to experimental conditions [4,5]. For instance, expression of commonly used RGs such as beta actin (*ACTB*), glyceraldehyde-3-phosphate dehydrogenase (*GAPDH*), 18S ribosomal RNA (*18S*), and hypoxanthine phosphor-ribosyl transferase (*HPRT*) is dependent on tissue type, developmental stage, and other factors [6–8]. In fact, the application of inappropriate RGs for normalization of the target gene could result in large fluctuations in expression among the tested samples, false conclusions, and misleading interpretations of gene expression [2,9]. Therefore, validation of the proper RGs for each experimental condition is considered as a prerequisite for reliable results by qPCR and can improve the accuracy and reproducibility of the study [5].

Because pigs (*Sus scrofa*) are one of the most economically important types of livestock, understanding how reproduction is regulated inherently or affected by external factors can offer important insights to aid in the development of husbandry strategies in the animal farm industry and understand the reproductive physiology of animals [10]. Normal reproductive function is dependent on the hypothalamic–pituitary–gonadal (HPG) axis; the HPG axis for reproduction is centrally controlled by a complex regulatory network of excitatory and inhibitory signals [11]. In detail, the hypothalamus secretes pulsatile gonadotropin-releasing hormone (GnRH) via the hypophyseal portal system to induce gonadotropin pulsatile secretion, including follicle-stimulating hormone and luteinizing hormone (LH), and the gonadotropins then stimulate the growth of ovarian follicles to preovulatory follicles, where estrogen is highly secreted. The estrogen activates the *GnRH* surge, which is followed by the LH surge for ovulation and corpus luteum (CL) formation. This inherent feedback system of the HPG axis can be altered by several stimuli, including disease, weight, nutrients, age, season, and stress, that can lead to loss of the normal preovulatory LH surge, estrus cyclicity, and fertility [11,12]. Therefore, gene expression study in HPG axis-related female tissues in response to internal or external stimuli has been widely conducted using qPCR [13,14].

Several studies have evaluated various RGs to clarify the most stable gene under each experimental condition. Similar efforts have also been conducted in pig to find the most stable RGs in various cell types, embryos, several tissues, and infection [2,4,5,7,15]. However, a clear list of suitable RGs in the HPG axis-related tissues in sows is still lacking. In addition, since the expression of genes in the reproductive system fluctuates depending on the stage of estrus cycle or pregnancy in animals, it is important to find the most stable RGs regardless of different stages of estrus cycle for the future experiment; application of other sets of RGs at each different stage of estrus cycle in a study is inconclusive and impractical [16]. Therefore, the aim of the present study was to evaluate stability within the pool of nine commonly used RGs in the HPG axis-related tissues of sows regardless of the stages of estrus cycle as transcript levels of RGs may vary among different types of tissues or between different estrus cycles. The cycle threshold (Ct) values determined by qPCR, which were obtained from candidate RGs in HPG axis-related tissues, were assessed for their stability by means of the geNorm and Normfinder programs. The RGs evaluated in the study will be helpful for investigating the molecular mechanisms involved in the HPG axis in female pigs.

## MATERIALS AND METHODS

### Ethics statement

All procedures for sampling animal specimens were approved by the Institutional Animal Care Use Committee at Kyungpook National University (approval number: 2021-0098).

### Chemicals and media

Unless otherwise specified, the chemicals and reagents were obtained from Thermo Fisher Scientific (Waltham, MA, USA).

### Acquisition of HPG axis-related tissues from sows

Samples from healthy individuals were collected after examination by two veterinarians. The HPG axis-related tissues were obtained from approximately two-year-old, threeway crossbred ([Landrace×Yorkshire]×Duroc sows; n = 12) that were not litter-mates, weighed approximately 200 kg, and had experienced 3 to 5 parities. The hypothalamus, which is located on the undersurface of the brain just below the thalamus, was bluntly dissected using micro forceps and snap-frozen into liquid nitrogen. The pituitary gland, which is located on the hypophysial fossa of the sphenoid bone and surrounded by the sella turcica, was also collected and snap-frozen. Both ovaries located alongside the lateral wall of the uterus were isolated; one was snap-frozen and the other was fixed with 4% paraformaldehyde (Duksan Chemical, Incheon, Korea) for determination of the estrus phase. The stage of estrus cycle in each sow was determined as the follicular or luteal phase when developing large follicles with degrading CLs or degrading small follicles with mature follicles were observable in the sectioned ovaries, respectively.

### Preparation of total RNA and cDNA and conducting qPCR

The extraction of total RNAs, preparation of cDNA, and qPCR runs to obtain Ct values were conducted according to a previously published article [9]. In brief, total RNA was extracted from HPG axis-related tissues using a QIA shredder column and RNeasy mini Kit (Qiagen, Hilden, Germany) with the RNase-free DNase treatment step for 15 min to eliminate residual genomic DNA. The concentration and purity of total RNA samples were quantified using an UV-Vis spectrophotometer (Nabi, MicroDigital Co., Ltd., Seongnam, Korea) via the A260/A280 ratio; pure total RNA samples within a 2±0.2 ratio were only selected fir further analysis. The cDNA was synthesized using 1 μg total RNA, 4 units Omniscript Reverse Transcriptase (Qiagen, Germany), 10 units RNase inhibitor, and 1 mM oligo dT primer at 60°C for 1 h using a thermal cycler (Qiagen, Germany). The qPCR was conducted using a Rotor Gene Q qPCR machine (Qiagen, Germany) with Rotor-Gene 2× SYBR Green mix (Qiagen, Germany), including 0.1 μg cDNA per reaction and 0.5 mM forward and reverse primers of RGs. The qPCR program consisted of predenaturation at 95°C for 10 min, 45 PCR cycles with 95°C for 10 s, 60°C for 6 s, and 72°C for 4 s, melting curve from 60°C to 95°C at 1°C/s, and cooling at 40°C for 30 s. Upon completing amplification by qPCR, the presence of gene-specific peaks with expected product size and the absence of primer dimers and nonspecific amplification were verified by melting curve analysis and electrophoresis using 1% agarose gel.

### Candidate RGs and their efficiency

During the qPCR assay, primers of nine commonly used RGs (*18S*, *HPRT1*, *GAPDH*, ribosomal protein 4 [*RPL4*], peptidylprolyl isomerase A [*PPIA*], beta-2-microglobulin [*B2M*], tyrosine 3-monooxygenase/tryptophan 5-monooxygenase activation protein, zeta polypeptide [*YWHAZ*], *ACTB*, and succinate dehydrogenase complex, subunit A [*SDHA*]) were selected on the basis of their different intracellular biological functions to avoid genes belonging to the same biological pathways that might be co-regulated in consideration of previous articles (Table 1) [2,5,7]. To validate the PCR efficiency of each RG, a standard curve of each primer of the RG was generated from Ct values obtained from a five-fold serial dilution set of cDNA (a mix of all cDNA samples) and calculated for parameters including slope, PCR efficiency (10^(1/–slope)^–1), and correlation (R^2^) by Excel (Microsoft, Redmond, WA, USA) in accordance with a previous article [2,7].

### Assessment of stability of reference genes in HPG axis-related tissues in sows

The stability rankings of the raw Ct values of each RG in the HPG axis-related tissues of sows were assessed by means of two well-known algorithms, geNorm and Normfinder [2,5,9]. The geNorm calculates gene expression stability (M value) from Ct values via the geometric average (normalization factors, NFs) of expression of RGs. During M value calculation, a gene determined as the highest M value is eliminated from the pool of RGs, and the new M value from the remaining RGs is then calculated continuously until the last two genes presenting the lowest M values are left, indicating the most stable RGs for that experimental condition. In addition, geNorm suggests the optimal number of RGs (NF_opt_) during the normalization step by investigating pairwise variation of stepwise inclusion between two sequential NF_n_ and NF_n+1_ (V_n/n+1_) from the two RGs with the lowest M value: the lowest value of V_n/n+1_ is determined as NF_opt_. Similar to geNorm, the average Ct value is converted to relative quantity data in Normfinder. Normfinder estimates intra and intergroup variation to determine the single most stable RG for which the lowest value corresponds to the most stability as well as the best combination of two RGs under experimental conditions.

### The application of normalization to RGs with different levels of stability

The effect of the most and least stable RGs was examined by normalization of the target gene against different RGs. The hypothalami were classified as preovulatory phase or luteal phase by observing developing follicles with Graafian follicles and degrading CLs or fully mature CLs in the sectioned ovaries, respectively. Thereafter, the hypothalamic gonadotrophin-releasing hormone (*GnRH*; Table 1) expression, the upstream regulator of HPG axis, as the target gene in different estrus phases was normalized against several RGs that were validated as the most or least stable in the present study.

### Statistical analysis

A one-way analysis of variance with Tukey’s post hoc test, Student’s *t*-test, or Pearson’s correlation between NF_opt_ and NF_3_ was applied using SPSS 12.0 (SPSS Inc., Chicago, IL, USA). Significant differences were considered at p<0.05.

## RESULTS

### Determination of the stage of estrus cycle in sows

By observing the sectioned ovaries (n = 12), the stages of estrus cycle of sows in the present study were classified as follicular phase showing large follicles with degrading CLs (n = 6) and luteal phase presenting degrading small follicles with mature follicles (n = 6). Since the present study was aimed to assess the most stable RGs in the HPG axis-related tissues regardless of the different stages of estrus cycle of sows, it was thought that the stages of estrus cycle of sows in the present study were evenly distributed for conducting further analysis.

### Primer specificity and efficiency to verify primers for the candidate RGs

**Figure 1:**
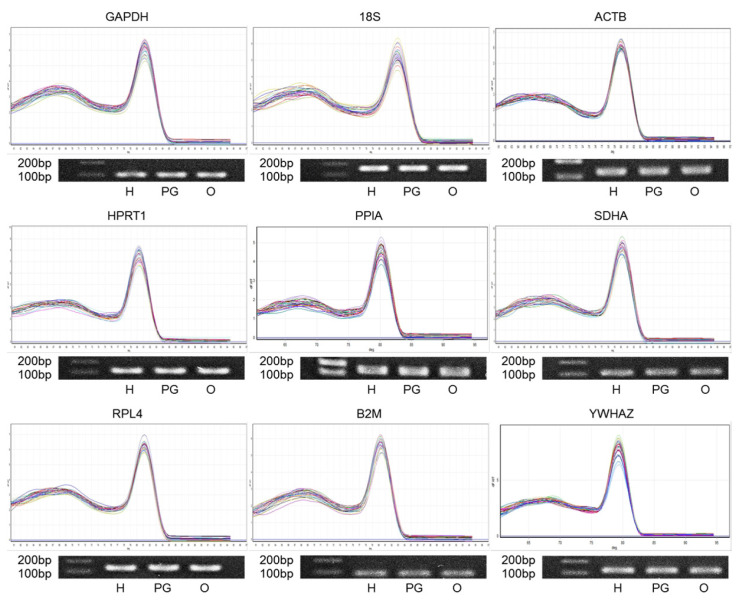
Confirmation of primer specificity and amplicon size in the primers for the candidate RGs. Each melting curve analysis of nine RGs is presented (top of individual genes), and each PCR product of amplification is shown by 1% agarose gel electrophoresis (bottom of individual genes). Lanes, which are displayed from the left to the right, show the 100 and 200 bp ladder, hypothalamus (H), pituitary gland (PG), and ovary (O). PCR, polymerase chain reaction; RG, reference genes.

The optical density ratio A260/A280 nm as measured with an UV-Vis spectrophotometer was 1.97±0.13 (ratio±standard deviation), indicating that the quality of total mRNA samples in the present study was suitable for the next analysis. The melting curve analysis was conducted at the end of the qPCR amplification program to assess the specificity of the nine RGs used in this study (Figure 1), with the result that all candidates of RGs were amplified with a high peak of single products and without any nonspecific amplification. Furthermore, appropriate PCR products with the expected size in Table 1 were determined by 1% agarose gel electrophoresis. The PCR efficiencies for the nine candidate RGs ranged between 0.95 and 1.03 (Table 2). According to these results, the present assay systems could be considered as valid for the quantification of transcripts and for further experiments.

### Average Ct values of RGs in HPG axis-related tissues

**Figure 2:**
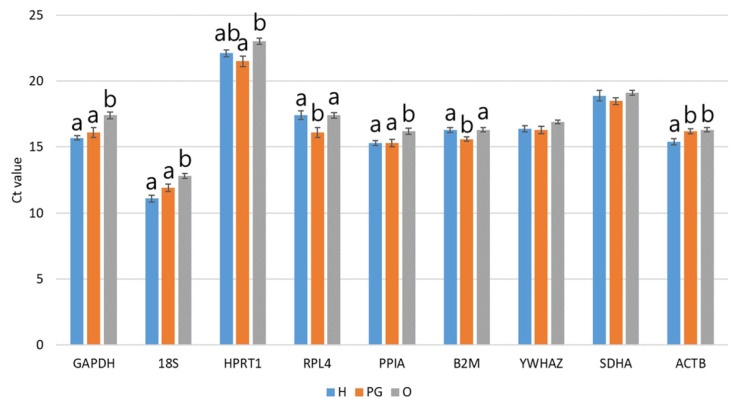
Average of Ct values in candidate RGs in HPG axis-related tissues. The Ct values of each primer were analyzed by ANOVA using Turkey’s post hoc test. Significant differences among H, PG, and O are indicated by different letters at the top of the bars (p<0.05). Graphs are presented as means±standard error of the mean. RG, reference genes; HPG, hypothalamic–pituitary–gonadal; ANOVA, analysis of variance; H, hypothalamus; PG, pituitary gland; O, ovary.

The mRNA transcription levels of nine RGs were directly compared by qPCR, and all amplifications were performed with an equal quantity of total RNA. The Ct values of seven of the nine candidate RGs showed significant differences (p<0.05) in the HPG axis-related tissues of sow (Figure 2). In particular, *GAPDH*, *18S*, *PPIA*, and *ACTB* exhibited significantly (p<0.05) higher Ct values in the ovary, indicating lower transcription levels in that location compared to the others. In contrast, *RPL4* and *B2M* showed significantly (p< 0.05) lower Ct values in the pituitary gland. These results indicate that the expression of RGs was influenced by the type of tissue, although RGs are believed to stably expressed.

### Assessment of stability in RGs using geNorm

**Figure 3:**
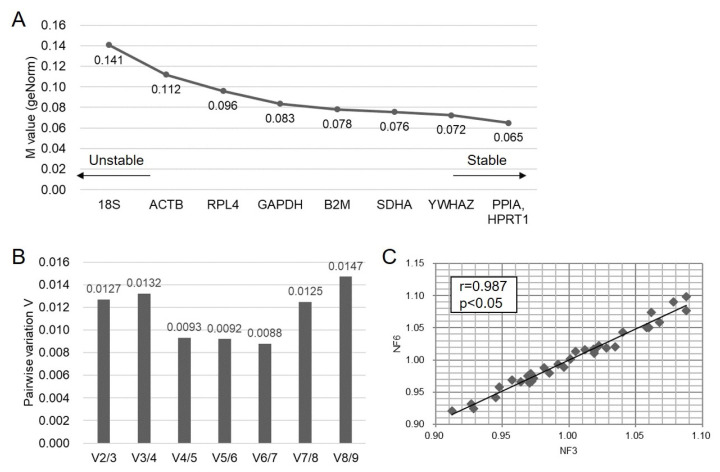
Ranking of stability (M-values) in candidate RGs by geNorm. M-values were analyzed from each Ct value of RGs of HPG axis-related tissues. The left side of the graph indicates high M-value, meaning low stability, and the right side of the graph presents high stability with low M-value (A). The optimal number of RGs is based on pairwise variation V_n/n+1_. The lowest V value (V_6/7_) indicates the optimal number of RGs during normalization to target genes (B). Pearson’s correlation analysis between NF_3_ and NF_opt_ (NF_6_) presents a linear regression with high correlation value (r = 0.987, p<0.05) (C). RG, reference genes; HPG, hypothalamic–pituitary–gonadal.

The raw Ct values of RGs in HPG axis-related tissues were assessed using the geNorm software program to rank their stability (M value; lower values were considered to be more stable) and identify the optimal number of RGs during normalization (V_n/n+1_; lower values have less variation) (Figure 3). *PPIA*, *HPRT1*, and *YWHAZ* were ranked as the three most stable genes in porcine HPG axis compared to the other RGs. The traditional RGs were assessed as least stable (*18S* and *ACTB*) or moderately stable (*GAPDH*) (Figure 3A). In addition, geNorm indicated that using six RGs (V_6/7_) in normalization was adequate in an assay of qPCR using HPG axis-related tissues of sows (Figure 3B). Since using an excessive number of RGs in an assay is inefficient and impractical when only a small number of target genes or rare samples are studied, we analyzed the correlation of NFs among the pool of the three most stable RGs (NF_3_), which corresponded to the three lowest M values, and optimal number of RGs (NF_6_ as NF_opt_) by Pearson’s correlation analysis. The result showed a high correlation between NF_3_ and NF_6_ (r = 0.987, p<0.05), indicating that the three most stable RGs in the present study were already sufficient for normalization when analyzing target genes in the HPG axis-related tissues (Figure 3C).

### Assessment of stability in RGs using Normfinder

**Figure 4:**
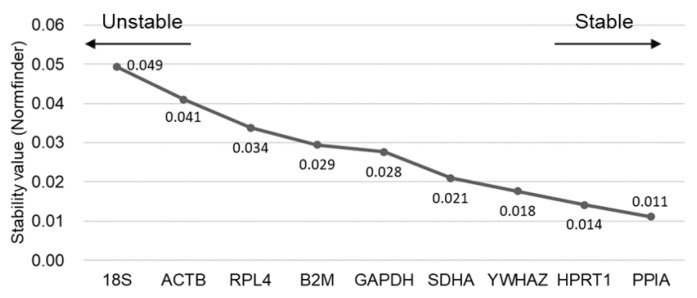
Values of stability of RGs by Normfinder analysis. Stability values of nine RGs were analyzed by estimating intra- and intergroup variation. The left and right side of the graph indicate low or high stability, respectively. RG, reference genes.

In the Normfinder analysis, the three most stable RGs in porcine HPG axis-related tissues were determined as *PPIA*, *HPRT1*, and *YWHAZ*, and the best combination of two genes from the nine candidates of RGs was revealed as *PPIA* and *HPRT1* (Figure 4). In contrast, two traditional RGs were assessed as the least stable (*18S* and *ACTB*). In short, there was good agreement in the stability results between geNorm and Normfinder programs. Therefore, we postulated the combination of *PPIA*, *HPRT1*, and *YWHAZ* to be the most suitable normalization approach to qPCR assay for HPG axis-related tissues in sows.

### Application to normalization of evaluated RGs to the target gene

**Figure 5:**
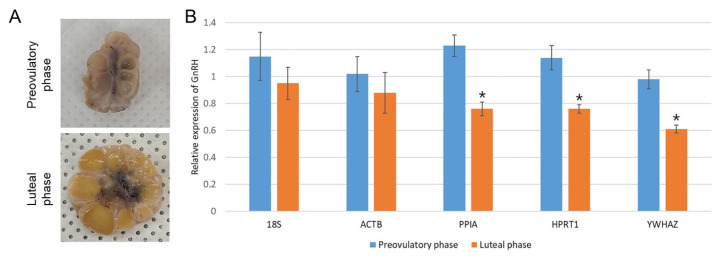
Application of different RGs to normalization of *GnRH* expression in the hypothalamus. The estrus cycles of sows were classified as preovulatory phase (dominant follicles and degrading CL) or luteal phase (fully mature CL) by observation of sectioned ovaries (A). The *GnRH* expressions in the hypothalamus under different estrus cycles were normalized against the most stable RGs (*PPIA*, *HPRT1*, and *YWHAZ*) and the least stable RGs (*18S* and *ACTB*). Significant (p<0.05) differences in *GnRH* expression level between preovulatory and luteal phase as determined by Student’s *t*-test are indicated with asterisks. Graphs are presented as means±standard error of the mean (B). RG, reference genes; *GnRH*, gonadotrophin-releasing hormone; CL, corpus luteum; *PPIA*, Peptidylprolyl isomerase A; *HPRT1*, hypoxanthine phosphoribosyltrasnfrase1; *YWHAZ*, tyrosine 3-monooxygenase/tryptophan 5-monooxygenase activation protein, zeta polypeptide; *ACTB*, beta actin.

Taken together, *PPIA*, *HPRT1*, and *YWHAZ* were found to be the three most stable RGs in the present study, whereas two traditional RGs (*18S* and *ACTB*) showed the least stability. Therefore, the effect of validated RGs under a particular experimental condition was examined by normalization to the target gene, *GnRH* (Figure 5). We classified the preovulatory phase and luteal phase by observation of sectioned ovaries (Figure 5A); thereafter, the corresponding hypothalami in the preovulatory and luteal phase (each n = 4) were selectively chosen to evaluate *GnRH* expression. In general, GnRH secretion in the hypothalamus is increased by elevated estrogen secretion from preovulatory follicles for LH surge during the late follicular (preovulatory) phase [14,17]. The hypothalamic *GnRH* expression as normalized against the three most stable RGs (*PPIA*, *HPRT1*, and *YWHAZ*) was significantly (p<0.05) elevated in the preovulatory phase compared to the luteal period (Figure 5B). In contrast, the traditional RGs (*18S* and *ACTB*), which were validated as unstable in HPG axis-related tissues, showed non-significance between the two estrus phases. These results suggest the importance of using proper and stable RGs that are specific to each experimental condition.

## DISCUSSION

An understanding of the HPG axis in the domestic animals is important in order to improve the output of livestock products as well as to understand animal reproductive disorders such as anestrus, silent heat, and ovarian cysts. In addition, its application can improve the efficiency of reproductive performance, including normal ovarian cyclicity, increased wean-to-estrus intervals, and higher pregnancy ratio and litter size [18]. Because gene expression studies using qPCR in HPG axis-related female tissues have been widely conducted to understand reproductive physiology, selection of adequate and stable RGs is regarded as a prerequisite step for deducing the reliable results for an exact comparison of mRNA transcription in different samples or tissues [8]. Unfortunately, there is no single universal RG that is constantly expressed in all types of tissues and is not regulated by internal and external stimuli [4]. And the expression of RGs in the reproductive tissues can be changed by inherent body conditions such as the stage of the estrus cycle or pregnancy in female pigs [16]. In addition, several previous articles and Figure 5 in the present study demonstrate that the application of stable or unstable RGs can change the outcome and conclusions of a study; for instance, whereas normalization with stable RGs could generate significant difference in target gene expression between groups, unstable ones showed no significance and possibly led to false conclusions [2,9]. Therefore, we mainly focused on uncovering the most stable RGs in the HPG axis-related tissues of sows regardless of the different stages of estrus cycle from the pool of nine commonly used RGs by means of stable RG-finding programs (geNorm and Normfinder). Since there has been no universal standard consensus method for the validation of stability of RGs, we mainly used geNorm for gene stability analysis, followed by reconfirmation by Normfinder to avoid tool-dependent results; both programs have been frequently used for finding stable RGs, and these comparisons by different programs for RG selection may allow a better evaluation of the most reliable controls [2,3,5,15,16, 19,20]. We found that results of the three most stable RGs were highly consistent between the two programs; a slight difference in stability rankings between programs could be explained by their different algorithms [2,19]. Comprehensively, both programs concluded that *PPIA*, *HPRT1*, and *YWHAZ* were the most stable RGs in the HPG axis in sows regardless of the stages of estrus cycle and that traditional RGs, including *18S* and *ACTB*, were less stable (Figures 3, 4). To the authors’ knowledge, the present study is the first to report on stable RGs in the HPG axis of pigs.

The present results explain the stability of RGs in HPG axis in sows; mainly, *PPIA*, *HPRT1*, *YWHAZ*, *18S*, and *ACTB*. Even though they are widely used as RGs due to their consistent roles in cell survival, their expression is affected by several stimuli. *PPIA* is a prototypical cyclophilin family member, an enzyme that catalyzes the reversible cis/trans interconversion of the imide bond in proline residues and is known as cyclosporin binding protein and inhibitor of serinethreonine phosphatase. Therefore, changes in expression of *PPIA* are highly related to inflammatory disorders and cancers [21]. *HPRT1* is a transferase enzyme that plays a pivotal role in the cell cycle by generating purine nucleotides via the purine salvage pathway, and its elevation is now considered as a marker with clinical significance in human disease, including several tumors, because demand for *HPRT1* in cell cycling is increased for nucleotide synthesis [22]. *YWHAZ* acts on cell growth, apoptosis, migration, and invasion. Furthermore, upregulation of *YWHAZ* is also highly related to tumor progression [23]. Because *ACTB* encodes a structural protein of cytoskeleton as an indispensable component of the cytoskeleton in the cell for cell migration, cell division, and regulation of gene expression, change of *ACTB* expression is associated with cancer and changes in response to external stimuli [9]. *18S* is a component of the ribosomal RNA and plays a role in the biogenesis and function of ribosomes in the cell; its expression is variable in cultured goat follicles and ovarian tissue derived from healthy or diseased humans [9]. Therefore, the fact that RGs could be affected by several influences makes the validation of RGs under each experimental condition important and suggests that random selection of commonly used RGs is no longer acceptable. In particular, several published articles have demonstrated differences in the transcript level (Ct value) of RGs. Ten of twelve RGs exhibited different Ct values in porcine mesenchymal stem cells (MSCs) before and after differentiation [5]. In addition, the Ct values of seven of nine RGs differed according to the type of porcine blastocysts [2]. Similarly, significant differences in Ct values among experimental groups have been routinely found [4,7,19–21]. In agreement with these articles, the present study found that the Ct values of seven of nine candidate RGs showed significant differences (p<0.05) in the HPG axis-related tissues of sows, indicating differences in tissue transcript levels even in RGs and suggesting the necessity of further validation of RGs for qPCR assay (Figure 2).

In porcine specimens, similar efforts to discover the most stable RGs in each experimental condition have been conducted. Expression of *YWHAZ* is the most stable RG in porcine MSCs regardless of differentiation induction [5], intact alveolar macrophages (AMs) [4], peripheral blood mononuclear cells (PBMCs) with lipopolysaccharide (LPS) and lipoteichoic acid (LTA) stimulation [19], tissue (PBMCs, lymph nodes, intestinal mucosa, stomach, liver, spleen, thymus, lung, kidney, heart, and skin) at different ages including newborn, young, and adult pigs [8], and adipose with muscle tissue [24]. *HPRT1* was also determined to be one of the best RGs in porcine samples for the pregnant ovary across physiological time points (heat and 15, 30, 45, and 60 days of pregnancy) [16], different parts of the gastrointestinal (GI) tract of piglets during the weaning process [22], and *Actinobacillus pleuropneumoniae*-infected tissues including white blood cells, liver, and lymph nodes [15]. *PPIA* is stably expressed in intact PBMCs, polyinosinic: polycytidylic acid-stimulated PBMCs [20], and several tissues at different ages [8]. In addition, in most of the aforementioned cases, traditional RGs such as *GAPDH*, *ACTB*, and/or *18S* were determined to be the least stable in porcine samples in porcine MSCs [5], pregnant ovary [16], intact AMs [4], LPS and LTA-stimulated PBMCs [19], the GI tract of piglets [22], heat-stressed blood [25], *Actinobacillus pleuropneumoniae*-infected tissues [15], and several tissues at different ages [8]. Furthermore, expression of these traditional RGs is unstable in several types of porcine blastocysts produced by *in vivo*, parthenogenetic activation, *in vitro* fertilization, and somatic cell nuclear transfer [2] and in various pig tissues including the diaphragm, heart, kidney, liver, lungs, muscle, spleen, and stomach [3,6,15].

Although there is currently no published data on stable RGs in HPG axis-related tissues of sows, similar studies have been conducted in other species. In agreement with the findings of the present study, *PPIA*, *HPRT1*, and/or *YWHAZ* have been validated as the most stable RGs in HPG-axis tissues including the brain, pituitary, ovary, and testis in songbirds [26], testosterone-influenced hypothalamus and kidney of rats [27], normal and adenoma tissues of pituitary gland from dogs and mice [28], and human polycystic ovarian syndrome [29]. The stability of traditional RGs such as *GAPDH*, *ACTB*, and/or *18S* was moderate to low in testosterone-influenced hypothalamus and kidney of rats [27], hypothalamus of chicken under different feeding status [30], HPG axis in songbird [26], pituitary gland from normal/adenoma dogs and mice [28], and human polycystic ovarian syndrome [29].

## CONCLUSION

Although ideal RGs should not be affected or regulated by experimental conditions, there is still no consensus on universal RGs that are perfectly constant. Therefore, because validation of RGs before normalization is considered to be an essential prerequisite step, we suggest that application of *PPIA*, *HPRT1*, and *YWHAZ* as RGs during qPCR can ensure more reliable results in the study of HPG axis in female pigs and avoid false or contradictory conclusions. These results may contribute to experimental design in the field of animal reproductive physiology as reference data.

## Figures and Tables

**Table 1:** Information on primers used in the present study

Gene name (symbol)	Primer sequences	Product (bp)	Accession number [Reference]
18S ribosomal RNA (*18S*)	F: tcgcggaaggatttaaagtg	141	NR_046261.1 [2,5]
	R: aaacggctaccacatccaag		
Beta-2-microglobulin (*B2M*)	F: tccgccccagattgaaattg	81	NM_213978.1 [5]
	R: tccttgctgaaagacaggtctg		
Peptidylprolyl isomerase A (*PPIA*)	F- aaaacttccgtgctctgagc	112	NM_214353.1 [2,5]
	R- ttatggcgtgtgaagtcacc		
Ribosomal protein 4 (*RPL4*)	F: caagagtaactacaaccttc	122	XM_005659862.3 [2,5,7]
	R: gaactctacgatgaatcttc		
Succinate dehydrogenase complex, subunit A (*SDHA*)	F: cacacgctttcctatgtcgatg	94	XM_021076931.1 [2,5]
	R: tggcacagtcagcttcattc		
Beta actin (*ACTB*)	F: tcaacaccccagccatgtac	84	XM_003124280.5 [2,5]
	R: agtccatcacgatgccagtg		
Glyceraldehyde-3-phosphate dehydrogenase (*GAPDH*)	F: acactcactcttctacctttg	90	NM_001206359.1 [2,5,7]
	R: caaattcattgtcgtaccag		
Hypoxanthine phosphoribosyltrasnfrase1 (*HPRT1*)	F: aagcttgctggtgaaaagga	100	NM_001032376.2 [2,5]
	R: gtcaagggcatagcctacca		
Tyrosine 3-monooxygenase/tryptophan 5-monooxygenase activation protein, zeta polypeptide (*YWHAZ*)	F: tgcttcctttgcttgcatcc	113	XM_001927228.7 [5]
	R: tcagggtaggcagggtttatag		
Gonadotrophin-releasing hormone (*GnRH*)	F: caacactggtcctatggattgc	186	NM_214274.1
	R: ctcttcaatcagactttccagagc		

**Table 2:** Information on correlation (R^2^) and PCR efficiencies of each RG

Gene	Correlation (R^2^)	PCR efficiencies
*18S*	0.991	0.99
*B2M*	0.993	1.00
*PPIA*	0.995	0.96
*RPL4*	0.989	0.97
*SDHA*	0.991	0.95
*ACTB*	0.988	1.01
*GAPDH*	0.993	0.99
*HPRT1*	0.995	1.03
*YWHAZ*	0.990	0.98

PCR, polymerase chain reaction; RG, reference genes; *18S*, 18S ribosomal RNA; *B2M*, Beta-2-microglobulin; *PPIA*, Peptidylprolyl isomerase A; *RPL4*, ribosomal protein 4; *SDHA*, succinate dehydrogenase complex, subunit A; *ACTB*, beta actin; *GAPDH*, glyceraldehyde-3-phosphate dehydrogenase; *HPRT1*, hypoxanthine phosphoribosyltrasnfrase1; *YWHAZ*, tyrosine 3-monooxygenase/tryptophan 5-monooxygenase activation protein, zeta polypeptide.
